# The Accuracy of Smart Devices for Measuring Physical Activity in Daily Life: Validation Study

**DOI:** 10.2196/10972

**Published:** 2018-12-13

**Authors:** Laurent Degroote, Ilse De Bourdeaudhuij, Maïté Verloigne, Louise Poppe, Geert Crombez

**Affiliations:** 1 Physical Activity & Health Department of Movement and Sports Sciences Ghent University Ghent Belgium; 2 Health Psychology Department of Experimental Clinical and Health Psychology Ghent University Ghent Belgium

**Keywords:** physical activity, fitness trackes, accelerometry

## Abstract

**Background:**

Wearables for monitoring physical activity (PA) are increasingly popular. These devices are not only used by consumers to monitor their own levels of PA but also by researchers to track the behavior of large samples. Consequently, it is important to explore how accurately PA can be tracked via these devices.

**Objectives:**

The aim of this study was, therefore, to investigate convergent validity of 3 Android Wear smartwatches—Polar M600 (Polar Electro Oy, Kempele, Finland), Huawei Watch (Huawei Technologies Co, Ltd, Shenzhen, Guangdong, China), Asus Zenwatch3 (AsusTek Computer Inc, Taipei, Taiwan)—and Fitbit Charge with an ActiGraph accelerometer for measuring steps and moderate to vigorous physical activity (MVPA) on both a day level and 15-min level.

**Methods:**

A free-living protocol was used in which 36 adults engaged in usual daily activities over 2 days while wearing 2 different wearables on the nondominant wrist and an ActiGraph GT3X+ accelerometer on the hip. Validity was evaluated on both levels by comparing each wearable with the ActiGraph GT3X+ accelerometer using correlations and Bland-Altman plots in IBM SPSS 24.0.

**Results:**

On a day level, all devices showed strong correlations (Spearman *r*=.757-.892) and good agreement (interclass correlation coefficient, ICC=.695-.885) for measuring steps, whereas moderate correlations (Spearman *r*=.557-.577) and low agreement (ICC=.377-.660) for measuring MVPA. Bland-Altman revealed a systematic overestimation of the wearables for measuring steps but a variation between over- and undercounting of MVPA. On a 15-min level, all devices showed strong correlations (Spearman *r*=.752-.917) and good agreement (ICC=.792-.887) for measuring steps, whereas weak correlations (Spearman *r*=.116-.208) and low agreement (ICC=.461-.577) for measuring MVPA. Bland-Altman revealed a systematic overestimation of the wearables for steps but under- or overestimation for MVPA depending on the device.

**Conclusions:**

In sum, all 4 consumer-level devices can be considered accurate step counters in free-living conditions. This study, however, provides evidence of systematic bias for all devices in measurement of MVPA. The results on a 15-min level also indicate that these devices are not sufficiently accurate to provide correct real-time feedback.

## Introduction

### Background

Physical inactivity is one of the major risk factors for mortality worldwide, causing an estimated 3.2 million deaths (6%) [1]. It accounts for approximately 21% to 25% of breast and colon cancers, 27% of type 2 diabetes, and 30% of burden because of ischemic heart disease [2,3]. It is hence recommended to perform a sufficient level of physical activity (PA). Physical activity is defined as “any bodily movement produced by skeletal muscles that require energy expenditure” [4]. PA can be classified according to the intensity of the activity using metabolic equivalents (METs). MET is the ratio of a person’s working metabolic rate relative to their resting metabolic rate. One MET is defined as the energy cost in rest and is equivalent to a caloric consumption of 1 kcal/kg/hour. It is estimated that compared with sitting, a person’s caloric consumption is more than 1.6 times higher and less than 3 times higher when being lightly active (1.6-3 METs), 3 to 6 times higher when being moderately active (3-6 METs), and more than 6 times higher when being vigorously active (>6 METs) [5]. Adults aged 18 to 64 years should accumulate at least 150 min of moderate-intensity aerobic PA throughout the week or do at least 75 min of vigorous-intensity aerobic PA throughout the week or an equivalent combination of moderate and vigorous intensity activity [5,6]. Another recommendation is to take at least 10,000 steps a day [7,8]. Nevertheless, 58% of the global population does not meet either of these recommendations [9].

Increasing the level of PA in the general population has proven notoriously difficult [10]. Scientists and practitioners have turned to behavior change theories to better understand the process of change and to better design interventions. Among various behavior change techniques, self-monitoring of the PA [11,12], has proven effective in changing PA levels. Consumer-level devices, also referred to as wearables, are increasingly used for the monitoring of PA [13]. They have built-in sensors to track and quantify daily movement [14].

Various wearables exist, and we can distinguish between activity trackers and smartwatches. Activity trackers (eg, Fitbit Flex, Misfit Shine, Garmin Vivosmart, and Xiaomi MiBand) are specifically built to track activity levels. Smartwatches (eg, Apple Watch, Samsung Gear, and Huawei Watch) also track activity levels but include other functions as well (eg, surfing the Web, receiving and answering mails or calls, playing music, and using the global positioning system). Furthermore, smartwatches allow downloading of apps and can be readily synchronized with a mobile phone. Smartwatches, therefore, have the potential to serve as a platform for app developers. They also have the potential to transform health care by supporting or evaluating health in everyday living because they (1) are familiar to most people; (2) are increasingly available as a consumer device; (3) enable near real-time continuous monitoring of PA and physiological measures; (4) support tailored messaging and reminders; (5) enable communication between patients, family members, and health care providers; and (6) allow for in situ mini-surveys and behavior verification based on sensor-based measure [15]. As wearables, both activity trackers and smartwatches, are increasingly popular not only with consumers but also with researchers [16], it is important to determine their accuracy for measuring PA variables such as step counts and minutes of MVPA.

Until now, only activity trackers have been scrutinized for their validity [17-23]. These studies found that most activity trackers (Fitbit Flex, Fitbit Zip, Fitbit One, Fitbit Charge HR, Jawbone Up, Nike+ Fuelband SE, Misfit Shine, and Withings Pulse) are valid for measuring steps but to a lesser extent, for measuring MVPA. For smartwatches, the validity for measuring PA variables (the number of steps and time spent in MVPA) has not been investigated. This is partly because of the recent rise in these devices: Up until 2014, about half of devices on the market were smartwatches. In 2015 and 2016, smartwatches represented 59.3% (143/241) of new devices on the market, whereas fitness trackers represented 40.7% (98/241) [24]. Furthermore, there is also a need for validation of wearables (both activity trackers and smartwatches) at a small time-scale. To our knowledge, all validation studies using activity trackers investigate validity on a daily level; however, validation using a smaller time-scale (eg, 15 min) is warranted. Increasingly, individual-focused interventions are developed that are based on real-time feedback. Examples are Just-In-Time adaptive interventions (JITAIs), which are the interventions that provide the right type and amount of support at the right time by adapting to an individual’s changing internal and contextual state. By providing this personally tailored support, interventions can be more effective in guiding users toward a physically active lifestyle [25]. Due to the internal sensors, the larger screen, and the fact that the device can be consulted constantly as they are worn on the wrist, smartwatches have the potential to serve as a platform for a JITAI. Notwithstanding the potential of smartwatches for JITAIs, smartwatches should be accurate in measuring physical active or inactive behavior during a short time duration [25,26].

For example, when users engage in a 15-min jog, the device has to be able to correctly categorize this behavior as 15 min of MVPA. On the basis of this measurement, the appropriate intervention component is to give real-time feedback to the user that he or she is doing well without giving other suggestions for more PA. However, when the user is not physically active for 15 min, the device has to be able to correctly categorize this as 15 min of physical inactivity. On the basis of this measurement, the appropriate intervention component is to provide real-time feedback in the form of a tailored suggestion to the user to engage in more PA.

### Objectives

The aim of this study was, therefore, to validate wearables in an adult population on both a day level as well as a 15-min level in free-living situations. We opted for a 15-min level because this is the smallest time level measured by the tested smartwatches. We opted for a validation in free living because this increases the external validity of our findings for use of wearables in daily life. We investigated convergent validity of 3 Android Wear smartwatches (Polar M600, Huawei Watch, and Asus Zenwatch3) and 1 activity tracker (Fitbit Charge). The number of steps and the time spent in MVPA measured by consumer-level devices was compared directly with the measurements of an ActiGraph GT3X+ accelerometer.

## Methods

### Participants

In this study, 36 healthy participants (50% male; mean age 39.43 years, SD 17.77) aged between 20 and 65 years and living in the area of Ghent (Belgium) were recruited using purposeful sampling. The inclusion criteria were having no current physical limitations, medical conditions, or psychiatric conditions. Before participants were selected, they completed the International Physical Activity Questionnaire (IPAQ, long 7d version) to assess their current level of PA. This procedure allowed us to have variation in the participants’ activity levels. The IPAQ was chosen for 2 reasons. First, a self-report measure was used for practical reasons. The self-report measurement allowed us to assess the current PA of people by letting them fill out a 10-min questionnaire, which makes it a very time-efficient measurement as opposed to objective measurement. Second, earlier research indicated that IPAQ is a reasonably reliable valid measurement tool for measuring habitual PA [27,28]. The International Physical Activity Questionnaire–Long Form (IPAQ-LF, last 7 days) asks participants to report the frequency and duration of activities in the last 7 days. Activities were classified into the domains of occupation, transportation, household, and leisure for each category of walking, moderate-intensity PA (MPA), and vigorous-intensity PA (VPA). Weekly and daily minutes of total PA, MPA, and VPA were computed.

On the basis of this assessment, we included 18 participants (50% male) who met the guideline of 30-min MVPA per day and 18 participants (50% male) who did not meet this guideline. All participants read and signed an informed consent form. The study protocol was approved by the ethics committee of the University hospital of Ghent (B670201731732).

### Instruments

#### Convergent Measure

The ActiGraph GT3X+ (Actigraph, Pensicola, FL, USA), a triaxial accelerometer was used as reference or convergent measure. The ActiGraph GT3X+ has been found to be reliable and valid. The GT3X+ is valid for measuring step counts compared with direct observation by trained observers [29-31] and for MVPA compared with indirect calorimetry [32,33]. Accelerometer data were initialized, downloaded, and processed by using ActiLife version 5.5.5-software (ActiGraph, Fort Walton Beach, FL, USA). The Freedson Adult (1998) cut-points were used to categorize PA measured by the ActiGraph accelerometer (sedentary activity=0-99 counts/min, light activity=100-1951 counts/min, moderate activity=1952-5723 counts/min, and vigorous activity ≥5724 counts/min) [32]. A 15-s epoch was used when downloading the data.

#### Wearables

We tested 4 wearables: Fitbit Charge, Polar M600, Huawei Watch, and Asus Zenwatch 3. Fitbits are one of the most popular activity trackers on the market. Smartwatches from Polar, Huawei, and Asus were selected because they use the Android Wear platform that has a significant market share (18% during Quarter 1 2017) and provides easy opportunities to program smartwatches and develop apps [34]. Polar M600, Huawei Watch, and Asus Zenwatch were selected because of their potential for electronic health interventions at the time of data collection (beginning of 2017). All 4 devices measure steps and a specific variable that quantifies the degree of PA. For the Fitbit, we used the variable *active minutes*, which is divided into light active, fairly active, and very active minutes. To approach the MVPA variable, fairly and very active minutes were summed. For the Android Wear smartwatches, we used the variable *active time*, which is calculated by summing the time spent on various activities (walking, running, and biking) that are all covered by the definition of MVPA (>3.0 MET) [1]. As all the devices set a goal of 30-min PA per day (similar to the MVPA recommendations for adults), we assumed that the measured variable corresponded to MVPA as measured by the ActiGraph. However, specific information regarding intensity cut-points is not publicly available. All Fitbit data were exported in an XLS (Microsoft Excel) format using the Fitbit Dashboard Web app. Every minute was categorized as sedentary, lightly active, fairly active, or very active. Afterward, the data per minute were converted to data per 15 min. Data from the Android Wear smartwatches were exported in a CSV (comma-separated values) format from Google Fit using Google Take Out. Every 15 min, it was shown how many seconds were spent on various activities (walking, running, biking, and tilting)

### Free-Living Protocol

As it was neither feasible nor comfortable to wear 4 wearables at the same time; participants were instructed to simultaneously wear 2 of the devices and the ActiGraph accelerometer for 2 consecutive days and then the other 2 wearables and the accelerometer for another 2 consecutive days. Between these 2 periods of 2 days, there was always a gap of 1 day on which devices were transferred from one participant to another. The devices were worn during all waking hours, except during water-based activities. All participants wore all 4 different wearables. All possible combinations of 2 wearables (a total of 6) were randomly assigned to the participants. Each combination was tested for 24 days in total, and each device was tested for 72 days. The ActiGraph GT3X+ was fitted to the right side of the participants’ waist, and the wearables were placed on the nondominant wrist. Furthermore, participants were instructed to keep a short diary in which they wrote down when they put on the devices and when and why they took them off.

### Statistical Analysis

Only days with valid data of the ActiGraph were included in the analysis. A valid day was defined as a 24-hour period in which at least 10 hours of data wear time was recorded. Nonwear time was analyzed as a run of zero counts lasting more than 60 min [35,36]. Analyses were performed using IBM SPSS Statistics version 24.0. All analyses were performed on a day level as well as a 15-min level. First, the correlation between the wearables and the ActiGraph accelerometer for measuring steps and MVPA was examined by calculating the Spearman *r* and ICC (absolute agreement, 2-way random, single measures, and 95% CI). Both analyses were conducted to take into account the possible systematic difference between the measurements, which is taken into account by the ICC, but not by the Spearman correlation. The following cut-off values were used to interpret the Spearman correlation: *r*<.20=very weak; .20 to .39=weak; .40 to .59=moderate; .60 to .79=strong; and .80 to 1.0=very strong [37]. The cut-off values to interpret the ICC were <.60=low; .60 to .75=moderate; .75 to .90=good; and >.90=excellent [38]. Second, to examine the level of agreement between the wearables and the convergent measure, Bland-Altman plots were constructed with their associated limits of agreement.

## Results

### Participants' Characteristics

Participants’ characteristics are presented in Table 1. All 36 participants wore the devices as planned. Some data were lost because of device malfunction (2 days MVPA or steps for Asus) and participant error such as not charging the device (4 days MVPA or steps for Asus, Polar, Fitbit, and Huawei). No data were lost from the ActiGraph GT3X+ accelerometers.

### Validation at a Day Level

In Table 2, the mean steps and mean minutes of MVPA (SD) per day are presented for all wearables and ActiGraph accelerometer. Moreover the statistical significance (*P* value) of the difference between the ActiGraph accelerometer and the wearables is presented. This table shows that every wearable overestimated the number of steps per day (not significant for Asus). For MVPA, Huawei, Asus, and Fitbit underestimated, whereas Polar overestimated the number of minutes of MVPA (not significant for Fitbit).

**Table 1:** Participant characteristics (N=36).

Characteristic	Minimum-maximum^a^	Mean (SD)^a^
Age (years)	20-65	39.43 (17.77)
Height (cm)	150-186	172.28 (8.22)
Weight (kg)	42-98	68.43 (12.09)
BMI^b^ (kg/m²)	17.51-32.00	23.00 (3.50)
MVPA^c^ (min/day)^a^	0-178.29	43.70 (42.02)

^a^Based on the International Physical Activity Questionnaire data.

^b^BMI: body mass index.

^c^MVPA: moderate to vigorous physical activity.

**Table 2:** Mean steps and minutes of moderate to vigorous physical activity per day measured by Huawei, Asus, Polar, and Fitbit and the corresponding ActiGraph measurements and statistical significance (*P* value) of the difference between the ActiGraph accelerometer and the wearables.

Variable		Wearable, mean (SD)/day	ActiGraph accelerometer, mean (SD)/day	*P* value
**Huawei**				
	Steps	8625 (4514)	7148 (3761)	.02
	MVPA^a^ (min)	27.24 (31.59)	36.97 (27.63)	.07
**Asus**				
	Steps	7662 (4380)	7082 (4148)	.42
	MVPA (min)	27.14 (33.18)	39.53 (36.33)	<.001
**Polar**				
	Steps	10,864 (7517)	7234 (4076)	<.001
	MVPA (min)	59.77 (62.94)	36.51 (28.31)	.03
**Fitbit**				
	Steps	9127 (5381)	7459 (3661)	.004
	MVPA (min)	35.47 (49.18)	41.98 (34.40)	.39

^a^MVPA: moderate to vigorous physical activity.

**Table 3:** Correlation coefficients, intraclass correlation coefficients, and 95% CI of the measurements at a day level.

Variable		Spearman *r* (95% CI)	ICC^a^ (95% CI)
**Huawei**			
	Steps	.892^b^ (0.779-0.930)	.885^b^ (0.822-0.926)
	MVPA^c^	.577^b^ (0.346-0.752)	.606^b^ (0.433-0.736)
**Asus**			
	Steps	.757^b^ (0.605-0.881)	.723^b^ (0.590-0.817)
	MVPA	.557^b^ (0.349-0.724)	.517^b^ (0.324-0.669)
**Polar**			
	Steps	.847^b^ (0.659-0.937)	.695^b^ (0.553-0.798)
	MVPA	.529^b^ (0.292-0.724)	.377^b^ (0.159-0.560)
**Fitbit**			
	Steps	.885^b^ (0.798-0.939)	.792^b^ (0.686-0.866)
	MVPA	.564^b^ (0.358-0.738)	.660^b^ (0.504-0.774)

^a^ICC: interclass correlation coefficient

^b^*P*<.001.

^c^MVPA: moderate to vigorous physical activity.

#### Correlations

**Figure 1:**
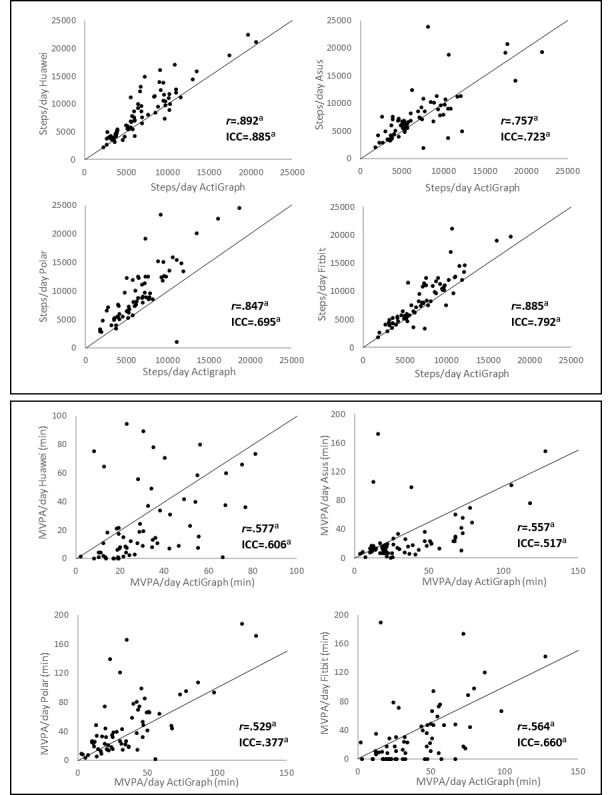
Correlations between the activity estimates per day from the wearables and the ActiGraph. Spearman *r* values and intraclass correlation coefficient values denote the correlation for measuring moderate-to-vigorous physical activity or steps between the wearable and the ActiGraph. a) P<.001. MVPA: moderate to vigorous physical activity; ICC: intraclass correlation coefficient.

For measuring steps on a day level, all wearables showed strong to very strong correlations based on the Spearman *r* and moderate to good agreement based on the ICC. Correlations between the MVPA levels from the wearables and the MVPA levels from the ActiGraph accelerometer were moderate based on the Spearman *r*. Agreements for MVPA between the wearables and the ActiGraph accelerometer were low. The correlation coefficients, ICC values, and associated 95% CI are shown in Table 3. The correlations are also illustrated in Figure 1. This figure shows that the scatter of the points around the line, reflecting the perfect agreement between measurements is larger for measuring MVPA than for measuring steps.

#### Level of Agreement

**Figure 2:**
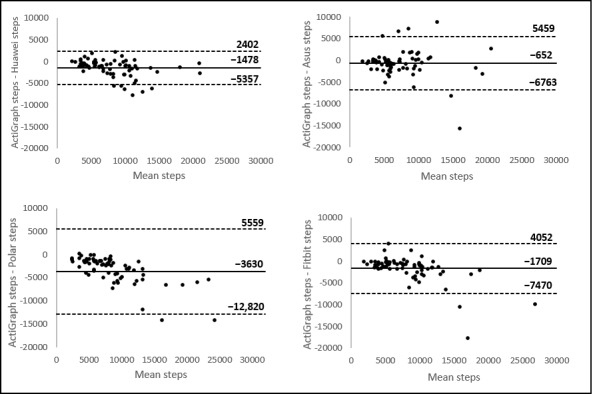
Bland-Altman plots of the wearables. The middle line shows the mean difference (Positive values indicate an underestimation of the wearable and negative values indicate an overestimation) between the measurements of steps of the wearables and the ActiGraph, and the dashed lines indicate the limits of agreement (1.96 × SD of the difference scores).

**Figure 3:**
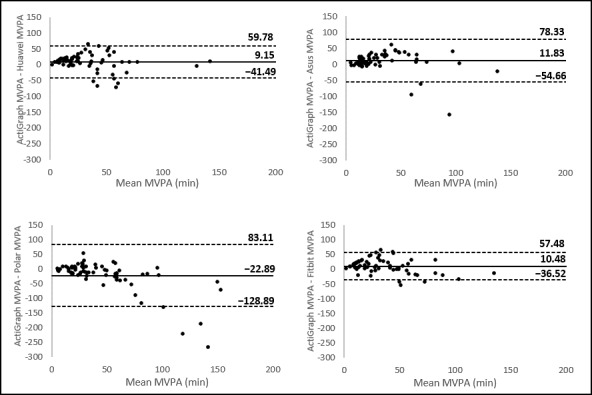
Bland-Altman plots of the consumer-level devices. The middle line shows the mean difference (Positive values indicate an underestimation of the consumer-level device and negative values indicate an overestimation) between the measurements of moderate-to-vigorous physical activity of the device and the ActiGraph, and the dashed lines indicate the limits of agreement (1.96 × SD of the difference scores). MVPA: moderate to vigorous physical activity.

Bland-Altman plots indicated the differences between the ActiGraph accelerometer and the wearables (y-axis) against the average number of steps or number of minutes of MVPA of the 2 devices (x-axis). Mean differences with the ActiGraph accelerometer and the limits of agreement for each wearable are presented in Figures 2 and 3. A positive value of the mean difference indicates an underestimation of the wearable compared with the golden standard, and a negative value indicates an overestimation. The systematic differences (mean differences) and the range between the upper and lower limits of agreement are important to make a statement about the validity of these wearables. The broader the range between the lower and the upper limit, the less accurate the measurements are. All wearables showed broad limits of agreement. For measuring steps, the plots (presented in Figure 2) showed the narrowest limits for Huawei (7759 steps) and the broadest limits for Polar (18,379 steps). The Bland-Altman plots for measuring MVPA are presented in Figure 3. For measuring MVPA, the narrowest limits were found for Fitbit (94 min), and the broadest limits were found for Polar (212 min).

### Validation at a 15-Minute Level

In Table 4, the mean steps per 15 min and mean minutes of MVPA per 15 min are presented for all devices. Moreover, the statistical significance (*P* value) of the difference between the measurements of the ActiGraph accelerometer and the wearables is presented. The results are displayed for (1) all 15-min time periods (including those with no MVPA) and (2) only the 15-min time periods in which MVPA was displayed with and without data revealing no MVPA. We opted to also present the latter to avoid distortion of the results. As users did not perform any PA during most periods of the day, a good agreement would be easy to obtain because of the many zero measurements by both measuring devices (wearable and ActiGraph accelerometer). In addition, this would reflect the validity of measuring physical inactivity rather than validity of measuring PA. Table 4 shows that every wearable device overestimated the number of steps per 15 min (all significant). For MVPA, Asus underestimated, whereas Huawei, Polar, and Fitbit overestimated the number of minutes of MVPA (not significant for Asus).

#### Correlation

**Figure 4:**
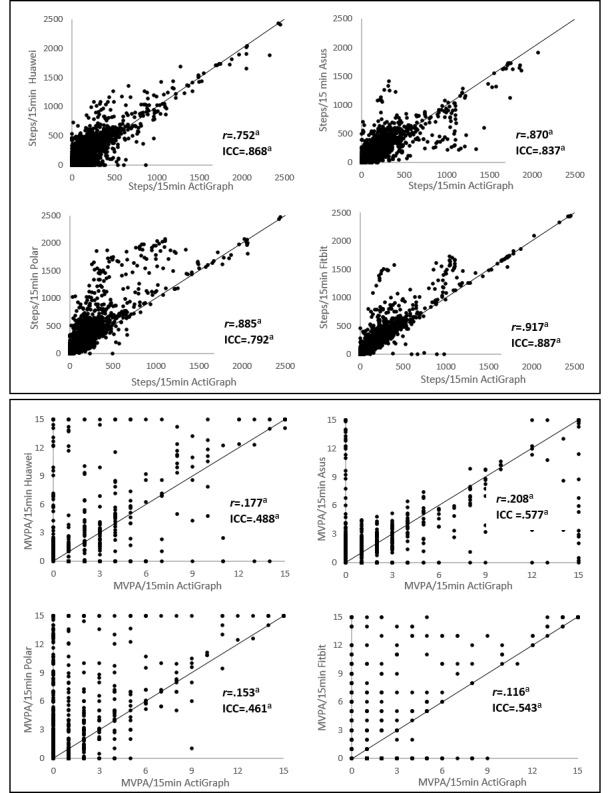
Correlations between the activity estimates per 15 min from the wearables and the ActiGraph GT3X+, Spearman *r* values, and intraclass correlation coefficient values that denote the correlation for measuring moderate to vigorous physical activity or steps between the wearables and the ActiGraph. a) P<.001. ICC: intraclass correlation coefficient; MVPA: moderate to vigorous physical activity.

**Figure 5:**
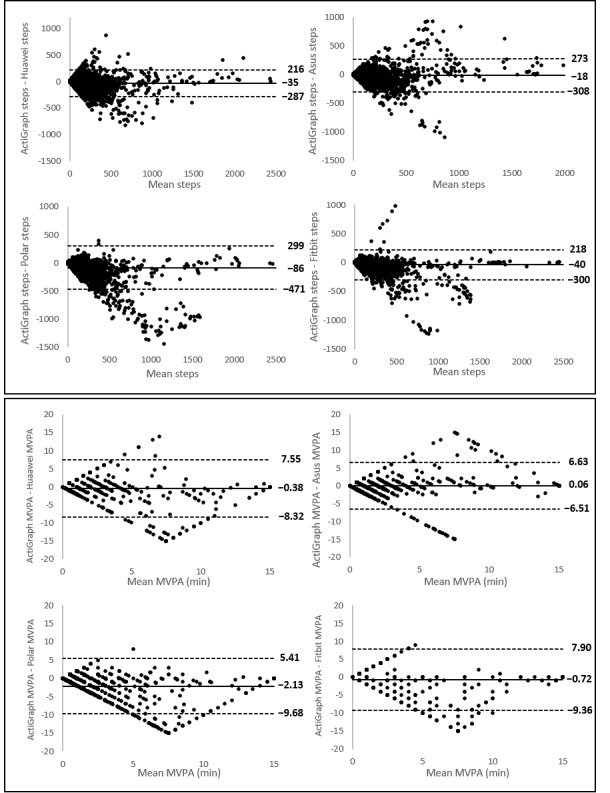
Bland-Altman plots of the wearables. The middle line shows the mean difference (positive values indicate an underestimation of the wearable and negative values indicate an overestimation) between the wearables and the ActiGraph, and the dashed lines indicate the limits of agreement (1.96 × SD of the difference scores). MVPA: moderate to vigorous physical activity.

All devices showed strong to very strong correlation based on the Spearman *r* and good agreement based on the ICC for measuring steps. For measuring MVPA (only including the data without zeros), correlations between readings from the wearables and the ActiGraph accelerometer were very weak to weak based on the Spearman *r*. Agreement between all the wearables and the ActiGraph accelerometer was low. The correlation coefficients, ICC values, and associated 95% CIs are shown in Table 5. The correlations are also illustrated in Figure 4. This figure revealed a systematic difference between the measurements of the wearables and the ActiGraph. The systematic difference increased as the number of steps or number of minutes MVPA increased. For example, an overestimation of 20% results in a difference of 200 steps on a day with 1000 steps. On a day, however, with 8000 steps, the difference between the measurements is 1600 steps. This is also evident from the Bland-Altman plot (Figure 5).

#### Level of Agreement

Mean differences with the ActiGraph accelerometer and the limits of agreement for each wearable device for measuring steps and MVPA are presented in Figure 5. For measuring steps, Huawei (503 steps) had the narrowest limits and Polar (770 steps) had the broadest limits. For MVPA, Asus (13.14 min) had the narrowest limits, and Fitbit (17.26 min) had the broadest limits.

**Table 4:** Mean steps and minutes of moderate to vigorous physical activity per 15 min measured by Huawei, Asus, Polar, and Fitbit and the corresponding ActiGraph measurements and statistical significance (*P* value) of the difference between the ActiGraph accelerometer and the wearables.

Variable		Wearable, mean (SD)/15 min	ActiGraph accelerometer, mean (SD)/15 min	*P* value
**Huawei**				
	Steps	184 (263)	148 (236)	<.001
	MVPA^a^ with zeros deleted (min)	2.91 (4.68)	2.53 (3.19)	.11
	MVPA (min)	0.86 (3.06)	0.54 (1.79)	<.001
**Asus**				
	Steps	166 (228)	147 (241)	.04
	MVPA with zeros deleted (min)	2.44 (3.62)	2.50 (3.67)	.76
	MVPA (min)	0.60 (2.07)	0.61 (2.12)	.76
**Polar**				
	Steps	231 (358)	145 (241)	<.001
	MVPA with zeros deleted (min)	3.75 (4.40)	1.62 (2.86)	<.001
	MVPA (min)	1.20 (3.03)	0.52 (1.78)	<.001
**Fitbit**				
	Steps	192 (304)	151 (247)	<.001
	MVPA with zeros deleted (min)	3.59 (5.28)	2.86 (3.82)	.003
	MVPA (min)	0.78 (2.86)	0.62 (2.13)	.01

^a^MVPA: moderate to vigorous physical activity.

**Table 5:** Correlation coefficients, intraclass correlation coefficients, and 95% CIs of measurements at a 15-min level.

Variable		Spearman *r* 95% CI	ICC^a^ 95% CI
**Huawei**			
	Steps	.752^b^ (0.728-0.772)	.868^b^ (0.859-0.877)
	MVPA^c^	.177^b^ (0.078-0.269)	.488^b^ (0.424-0.547)
**Asus**			
	Steps	.870^b^ (0.851-0.880)	.837^b^ (0.825-0.847)
	MVPA	.208^b^ (0.118-0.304)	.577^b^ (0.524-0.625)
**Polar**			
	Steps	.885^b^ (0.875-0.898)	.792^b^ (0.778-0.806)
	MVPA	.153^b^ (0.080-0.223)	.461^b^ (0.408-0.512)
**Fitbit**			
	Steps	.917^b^ (0.906-0.928)	.887^b^ (0.879-0.895)
	MVPA	.116^b^ (0.007-0.223)	.543^b^ (0.481-0.599)

^a^ICC: interclass correlation coefficient

^b^*P*<.001.

^c^MVPA: moderate to vigorous physical activity.

## Discussion

### Principal Findings

This study investigated the validity of 4 wearables (3 smartwatches and 1 activity tracker) for measuring steps and MVPA in naturalistic situations. Validity was investigated separately for a day level and a 15-min level. The ActiGraph GT3X+ accelerometer was used as a convergent measure. The results can be readily summarized.

First, all 4 wearables showed good validity for measuring steps on a day level and a 15-min level. Nevertheless, all devices overestimated the number of steps. Second, for estimating MVPA, our study results demonstrated systematic bias for all wearables, both on a day level and a 15-min level, suggesting the validity is moderate to low for MVPA.

Although we cannot compare the overestimations of the steps per day for the smartwatches with previous studies, an overestimation for Fitbit has been reported before [18,35,39]. These studies showed that Fitbit overestimated steps on average by about 4% to 13% per day (step difference between wearable and Actigraph/steps measurement of the ActiGraph), which is a smaller overestimation than what we found. The overestimation for Fitbit (on average 1709/9126 steps, 18.72%), Huawei (on average 1477/8626 steps, 17.12%), and Polar (on average 3630/10,854 steps, 33.44%) was substantially larger. The overestimation on a day level was the smallest for Asus (on average 652 on 7662 steps; 8.50%). Moreover, on a 15-min level, all 4 devices overestimated the amount of steps: Huawei with on average 19.0% (35/184 steps), Asus with on average 10.8% (18/166 steps), Polar with 37.2% (86/231 steps), and Fitbit with 21.2% (41/193 steps). When looking at the limits of agreement on both levels, Polar shows the broadest limits, whereas Huawei shows the smallest limits. From this, it can be concluded that Polar is the least accurate device for measuring steps and that, despite the smallest mean difference being that of Asus, Huawei is the most accurate device for measuring steps. There are several reasons that may account for the systematic overestimation. First, the overestimation may also be explained by the different wear location of the devices. The ActiGraph GT3X+ is worn on the hip, whereas the wearables are worn on the wrist. This by itself could result in different measurements. Previous research concluded that wrist attachment devices detected consistently fewer counted steps than the waist attachment devices at most treadmill speeds during laboratory testing. In contrast, wrist attachment devices detected a higher average step count than the waist attachment devices under free-living conditions [40]. Second, the overestimation may also be explained by the algorithms used to convert raw activity data from the different sensors in the watches into steps. Companies may use a lower threshold for steps than the threshold for the ActiGraph accelerometer algorithm. In line with this hypothesis, the systematic error increased as the number of steps increased.

All devices displayed information on how much time per day was spent in PA of at least moderate intensity. In contrast to measuring steps, wearables showed only moderate validity for measuring MVPA relative to the ActiGraph GT3X+ accelerometer on a day level and even low validity on a 15-min level. Whether MVPA was overestimated or underestimated varied depending on the device type and the time level. On a day level, Fitbit, Huawei, and Asus underestimated MVPA with an average of 30% (10/35 min per day), 16% (9/57 min per day), and 36% (12/33 min per day), respectively, whereas Polar overestimated MVPA with 33% (23/70 min per day). When looking at the limits of agreement on a day level, Fitbit shows the narrowest limits, whereas Polar shows the broadest limits. Moreover, Huawei shows rather narrow limits, making it, in combination with the small mean difference, the most accurate for measuring MVPA on a day level. Polar, however, is the least accurate. On a 15-min level, Fitbit, Huawei, and Polar overestimated MVPA with 20% (0.72/3.60 min), 13% (0.38/2.91 min), and 57% (2.13/3.75 min), respectively, whereas Asus underestimated MVPA with 2% (0.06/2.44 min). Asus also showed the narrowest limits of agreement, meaning it is the most accurate wearable device for measuring MVPA on a 15-min level. The results of Fitbit Charge on a day level are in line with the findings of a validation study of Fitbit Flex in naturalistic settings in which an underestimation of 36% time spent on MVPA per day was found [21]. Other studies in naturalistic settings found an overestimation of the MVPA measurements by Fitbit on a day level with 77% to 153% per day [19,41]; however, in these studies, Fitbit was worn on the hip. The difference between the findings of these previous studies and this study can, therefore, be explained by the placement of the wearable. Ferguson et al and Reid et al investigated the validity of Fitbit One, Fitbit Zip, and Fitbit Flex. All these wearables are worn on the hip.

A possible explanation for the moderate to low validity found in our study could be that the PA variables measured by the devices were not explicitly identified as MVPA. However, because all devices had set a goal of 30 min PA per day (similar to the MVPA recommendations for adults), we assumed that the measured variable corresponded to MVPA as measured by the ActiGraph accelerometer. Nevertheless, specific information regarding intensity cut-points was not provided and publicly available from these 4 wearables. An earlier study showed that using different intensity cut-points in accelerometers resulted in different MVPA levels [42], suggesting that it is difficult to compare accelerometer MVPA measurements when intensity cut-points vary. This could be the case in this study, which makes it difficult to compare the Actigraph accelerometer MVPA measurements with the wearable MVPA measurements [43]. However, our results showed large inconsistent underestimations and overestimations between and within participants, which cannot only be attributed to the lack of definitional similarity of the measured variable. Therefore, the discrepancies here may be a result of both definitional and measurement problems (eg, sensitivity algorithm). These findings are in line with previous studies that have expressed concerns that such devices might not be able to provide adequate information to guide exercise intensity or detect MVPA [17].

The inclusion of 4 popular devices enables to draw conclusions on the validity of these 4 smartwatches and not only on a singular device. Moreover, to the best of our knowledge, this was the first study to explore validity of smartwatches to measure steps and MVPA. The key strength of this study is the validation of the wearables on a 15-min level to investigate the potential of the devices to correctly situate physically active behavior over time to provide exact real-time feedback on PA behavior. Despite the clear results of this study, it is important to see them in the context of the purpose of the devices. The main purpose of these devices is to motivate the user to move more in everyday life, suggesting that 100% accurate measurements might not be needed. Modest accuracy can be good enough for this purpose [44]. Furthermore, this study has some other limitations. First, the choice of a 15-min level is arbitrary. It was the smallest data collection window in the Android Wear smartwatches. Ideally, validation on a smaller time-level, such as 1 or 5 min, should be performed to be able to better estimate the potential for providing real-time feedback. However, we can, based on the 15-min timescale, assume that these wearables will logically also not be accurately measuring MVPA on a smaller time-scale (eg, 10 min, 5 min, 1 min, and 30 s). Second, we used the ActiGraph accelerometer as convergent measure and not as a criterion measure, meaning it may not be considered the true golden standard. Although earlier studies showed good validity of the ActiGraph GT3X+ for measuring MVPA compared with indirect calorimetry, the main limitation for both uniaxial and triaxial accelerometers is the inability to accurately assess the movement associated with nonambulatory activity, such as cycling, especially with hip-worn devices [45]. For measuring steps, the golden standard is direct observation. For measuring MVPA, which is a complex and multifaceted construct, there is currently no consensus [46,47]. As by definition, PA leads to energy expenditure; the doubly labeled water (DLW) method, which assesses total energy expenditure over longer periods of time, is the golden standard to assess physical activities in laboratory settings [47,48]. However, because of feasibility, direct observation and DLW are impossible in free-living conditions. The ActiGraph was, therefore, by approximation, the best available golden standard. Third, the sample size was small but comparable with previous validation studies [19-21,38,41,49]. Fourth, the development of new wearables that appear on the market is going fast. Therefore, the need for further validation in naturalistic settings remains. Obviously, it is not possible to validate each single new device coming onto the market. However, we must always remain critical of measurements of PA by new devices, and research must continue to invest resources and time in this type of research, especially when new devices also have potential to be used within research. In this respect, it may be very useful in the future when manufacturers provide more insight into the cut-points and algorithms that were used to translate the raw data into useful information (such as steps and minutes of MVPA).

### Conclusions

Generally, it can be concluded that all 4 consumer-level devices (Huawei Watch, Polar M600, Asus ZenWatch2, and Fitbit Charge) are valid devices to estimate the amount of steps in naturalistic situations on both a day level and 15-min level. Nevertheless, for estimating MVPA, our study reveals systematic bias for all devices, both on a day level and a 15-min level, suggesting the validity is moderate to low for MVPA. This suggests that these wearables cannot replace the current generation of research-based accelerometers such as the ActiGraph GT3X+ to assess MVPA. The MVPA results on a 15-min level also indicate that these devices are not accurate in giving feedback on how many minutes the user performed MVPA in the past 15 min. Although we were not able to investigate validity on a smaller time-scale, we can, based on the 15-min time-scale, assume that these wearables will not be accurate in measuring MVPA on a smaller time-scale as well (eg, 10 min, 5 min, 1 min, 30 s). Consequently, these wearables cannot be considered to have the potential to provide exact real-time feedback on minutes MVPA. Therefore, we conclude that these wearables cannot be used to inform the design of a JITAI or to serve as a platform for a JITAI to increase PA levels.

## Abbreviations

DLW: doubly labeled water
ICC: interclass correlation coefficient
IPAQ: International Physical Activity Questionnaire
JITAI: Just-In-Time adaptive intervention
METs: Metabolic Equivalents
MPA: moderate-intensity physical activity
MVPA: moderate to vigorous physical activity
PA: physical activity
VPA: vigorous-intensity physical activity
